# Cholecystokinin Activation of Cholecystokinin 1 Receptors: a Purkinje Cell Neuroprotective Pathway

**DOI:** 10.1007/s12311-022-01428-x

**Published:** 2022-06-23

**Authors:** Harry T. Orr

**Affiliations:** 1 Institute of Translational Neuroscience, University of Minnesota, Minneapolis, MN 55455 USA; 2 Department of Laboratory Medicine and Pathology, University of Minnesota, Minneapolis, MN 55455 USA

**Keywords:** Spinocerebellar ataxia, Purkinje cells, Cholecystokinin, mTORC1 signaling, Neuroprotection

## Abstract

This is a summary of the virtual presentation given at the 2021 meeting of the Society for Research on the Cerebellum and Ataxias, https://www.meetings.be/SRCA2021/, where the therapeutic potential of the CCK-CCK1R pathway for treating diseases involving Purkinje cell degeneration was presented. Spinocerebellar ataxia type 1 (SCA1) is one of a group of almost 50 genetic diseases characterized by the degeneration of cerebellar Purkinje cells. The SCA1 *Pcp2-ATXN1[30Q]D776* mouse model displays ataxia, i.e. Purkinje cell dysfunction, but lacks progressive Purkinje cell degeneration. RNA-seq revealed increased expression of cholecystokinin (CCK) in cerebella of *Pcp2-ATXN1[30Q]D776* mice. Importantly, the absence of Cck1 receptor (CCK1R) in *Pcp2-ATXN1[30Q]D776* mice conferred a progressive degenerative disease with Purkinje cell loss. Administration of a CCK1R agonist to *Pcp2-AXTN1[82Q]* mice reduced Purkinje cell pathology and associated deficits in motor performance. In addition, administration of the CCK1R agonist improved motor performance of *Pcp2-ATXN2[127Q]* SCA2 mice. Furthermore, CCK1R activation corrected mTORC1 signaling and improved the expression of calbindin in the cerebella of *AXTN1[82Q]* and *ATXN2[127Q]* mice. These results support the Cck-Cck1R pathway is a potential therapeutic target for the treatment of diseases involving Purkinje neuron degeneration.

## Introduction

The Spinocerebellar ataxias (SCAs) are a genetically heterogeneous group of over 45 autosomal dominant neurodegenerative diseases. In many SCAs, dysfunction along with degeneration of cerebellar Purkinje cells precedes pathology in other neuronal cells/regions [1, 2]. This feature of the SCAs supports the concept that Purkinje cells are more vulnerable to cellular perturbations than other neurons [3–5].

Numerous unique features of Purkinje cells very likely underlie their enhanced vulnerability to perturbations. Purkinje cells are large neurons with an extensive and elaborate dendritic tree that has a large excitatory synaptic input. For example, climbing fiber synapses onto Purkinje cells, which generate complex spike excitatory postsynaptic potentials, are among the most powerful excitatory synapses in the brain. In addition, Purkinje cells generate autonomous pacemaking spikes. Consistent with these electrophysiological and structural properties, Purkinje cells have remarkably high metabolic activity. Furthermore, as noted by Hekman and Gomez (2015), several reports demonstrate that disruptions in protein homeostasis impact Purkinje cells before other neurons in mice and humans [6–10]. This aspect of Purkinje cell vulnerability is particularly relevant to SCAs as deficits in protein homeostasis are a recurring pathogenic theme for these diseases [11].

The utilization of SCA mouse models has been critical for gaining insight into the molecular basis of these disorders [5]. In the case of Purkinje cell vulnerability in SCA1, cerebellar transcriptomic analyses were used for the identification of both disease progression and protection pathways [12]. Notably, it was found that the level of cholecystokinin *(CCKs)* expression and its subsequent interaction with the CCK1 receptor is a potential Purkinje cell protective pathway in SCA1 mice. Subsequently, it was demonstrated that administration of a Cck1R agonist improves motor performance, corrects mTORC1 signaling, and improves the expression of calbindin in the cerebella of SCA1 and SCA2 transgenic mice. These results indicate that manipulation of the CCK-CCK1R pathway is a potential therapeutic target for the treatment of diseases involving Purkinje neuron degeneration. This minireview discusses the evidence supporting the CCK-CCK1R as a protective pathway in SCA mice and speculates on the broader implications of these findings.

## Purkinje Cell Protection by CCK is Dependent on Presence of CCK1 Receptor

**Fig. 1:**
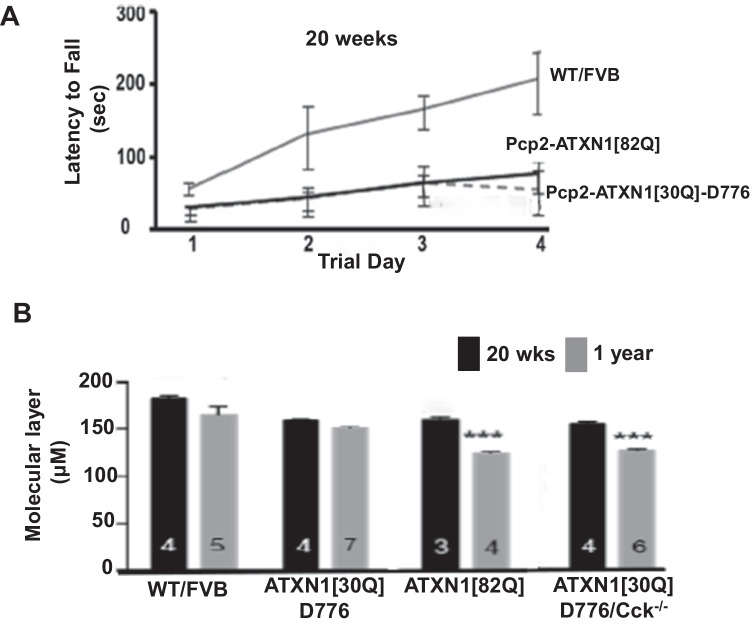
Elevated CCK expression is protective against progressive Purkinje cell atrophy in *ATXN1[30Q]D776* mice. **A** Four-day trial of accelerating Rotarod performance of ATXN1 transgenic mice at 20 weeks of age. Both *ATXN1[30Q]-D776* (*n* = 3) mice and *ATXN1[82]-S776* (*n* = 4) at 20 weeks of age were compared to age-matched WT/FVB (*n* = 3) (*p* = 0.04 and *p* = 0.03) ± SEM. **B** Cerebellar molecular layer thickness in *ATXN1* transgenic mice at 20 weeks of age and 1 year of age. *N*’s are indicated inside the bars for each genotype. ***p < 0.001

The CCK-CCK1R line of investigation began with a study to examine the role of ATXN1-S776 phosphorylation in Purkinje cell SCA1 pathogenesis. It was found that replacing the serine amino acid with a potential phospho-mimicking aspartic acid at position 776 transformed wild type (WT) ATXN1[30Q] into a pathogenic protein. Importantly, while mice expressing *ATXN1[30Q]-D776* in Purkinje cells manifested severe ataxia from an early age, i.e. as severe as mice with Purkinje cell expression of *ATXN1[82Q]*, disease in *ATXN1[30Q]-D776* mice was not progressive leading to Purkinje cell degeneration, i.e. atrophy of the molecular layer, as was observed in *ATXN1[82Q]* mice (Fig. 1A and B and [13]). Reasoning that perhaps the failure of Purkinje cell pathology to progress in *ATXN1[30Q]D776* mice is due to activation of a neuroprotective pathway(s), it was hypothesized that activation of a protective pathway might elevate expression of critical gene(s) in *ATXN1[30Q]D776* cerebellar RNA relative to WT and *ATXN1[82Q]* cerebellar RNA. An RNA-seq analysis revealed that *CCKs* expression was much higher in *ATXN1[30Q]D776* compared to *ATXN1[82Q]* cerebella [12]. Moreover, absence of either CCK (Fig. 1B) or CCK1R in *ATXN1[30Q]D776* mice [12] resulted in a Purkinje cell disease that is as progressive as in *ATXN1[82Q]* transgenic mice as assessed by atrophy of the molecular layer and loss of Purkinje cells.

Cholecystokinin (CCK), originally discovered in the gastrointestinal tract and typically associated with regulation of food intake and satiation, is one of the most abundant neuropeptides in the brain [14]. Within the cerebellum, *CCK* is predominately if not exclusively expressed by Purkinje cells [15, 16], a point further supported by ISH data from the Allen Brain Atlas [https://portal.brain-map.org/]. Cck effects are mediated by two G-protein coupled receptors [14], CCK1R and CCK2R (first designated as Cckar and Cckbr), and CCK1R is expressed in the adult mouse cerebellum in a Purkinje cell-enriched manner (Allen Brain Atlas). Based on the Purkinje cell protective effects of CCK in *ATXN1[30Q]D776* mice, we speculated that activation of CCK1R would be protective to Purkinje cells expressing ATXN1 with an expanded polyglutamine tract.

## CCK1R Activation in ATXN1[82Q] and ATXN2[127] Transgenic Mice: Restoration of Purkinje Cell Homeostasis and mTORC1 Signaling

A71623, a CCK1R tetrapeptide agonist, is highly selective for CCK1R [17]. In rodents, peripheral administration elicits CNS-mediated behavioral effects [18, 19]. To examine further the protective capability of CCK1R activation in SCA Purkinje cells, we assessed the ability of this CCK1R agonist to improve Purkinje cell function in transgenic mouse models of SCA1 and SCA2 [20]. In these transgenic mice, the expanded ATXNs were expressed specifically in Purkinje cells using the *Pcp2* regulatory region [21, 22].

**Fig. 2:**
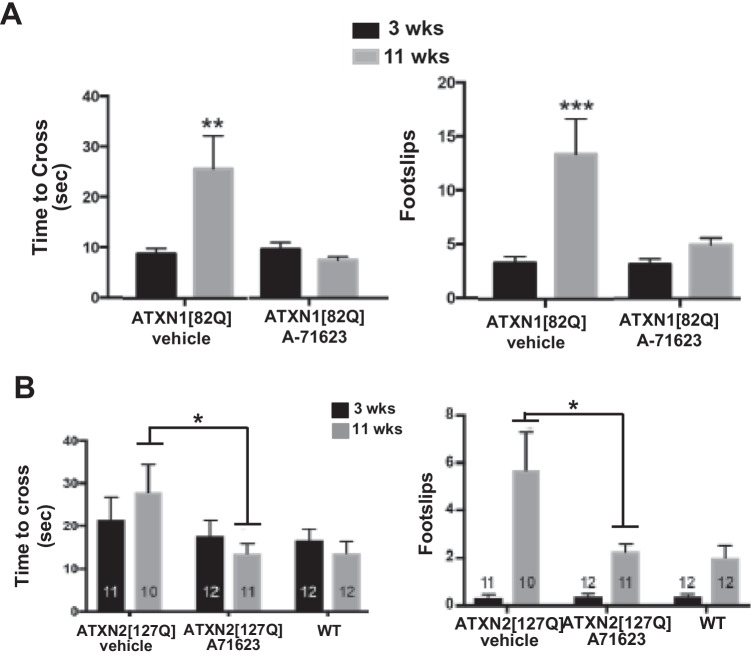
CCK1R agonist A71623 improves motor performance in *ATXN1[82Q]* and *ATXN2[127Q]* mice. Mice were tested for motor performance using the bar cross at 3 weeks of age. Then either 0.02 mg/kg/day A71623 or vehicle (20 mM PBS) was given and mice were retested on bar cross at 11 weeks of age. **A** Time to cross and the number of foot slips on the 10 mm round balance beam for *ATXN1[28Q]*. **B** Time to cross and the number of foot slips on the 10 mm round balance beam for *ATXN2[127Q]*. N’s are indicated inside each bar for each genotype/test. Error bars are SEMs. Two-way ANOVA with Tukey post hoc test, *p < 0.05, **p < 0.01, ***p < 0.001

Administration of the CCK1R-selective agonist A71623 mitigates motor performance deficits in both *Pcp2*-*ATXN1[82Q]* and *Pcp2*-*ATXN2[127Q]* mice (Fig. 2). Consistent with A71623 improving Purkinje cell function, A71623 also increased expression of calbindin, a molecular marker of Purkinje cell health [23], in *Pcp2*-*ATXN1[82Q]* and *Pcp2*-*ATXN2[127Q]* mice. These results provide direct evidence that activation of CCK1Rs is protective to Purkinje cells in SCA1 and SCA2.

Activation of CCK1R is known to impact a wide variety of signaling pathways [24]. Interestingly, among the pathways affected by CCK1R activation is mTORC1, and mTORC1 signaling contributes to Purkinje cell dysfunction in *Atxn1*^*154Q/2Q*^ knock-in mice [25]. The status of mTORC1 was assessed in cerebella from *Pcp2*-*ATXN1[82Q]* and *Pcp2*-*ATXN2[127Q]* mice by measuring the levels of phosphorylated ribosomal protein S6 (pS6) and phosphorylated translational repressor 4e-bp1 (p4e-bp10), both targets of mTORC1 [26]. Though mTORC1 was altered in both *Pcp2*-*ATXN1[82Q]* and *Pcp2*-*ATXN2[127Q]* mice, intriguingly, it was decreased in *Pcp2*-*ATXN1[82Q]* mice and increased *Pcp2*-*ATXN2[127Q]* mice. Yet, the CCK1R agonist A71623 normalized mTORC1 cerebellar signaling in both *Pcp2*-*ATXN1[82Q]* and *Pcp2*-*ATXN2[127Q]* mice.

## Discussion

Data reviewed here support activation of Purkinje cell CCK1Rs as being protective to these neurons in *Pcp2*-*ATXN1[82Q]* and *Pcp2*-*ATXN2[127Q]* transgenic mice. A critical aspect of the protective effects of CCK1R activation is a subsequent restoration of normal mTORC1 signaling whether mTORC1 signaling is decreased, as in *Pcp2*-*ATXN1[82Q]* Purkinje cells, or enhanced, as seen in *Pcp2*-*ATXN2[127Q]* Purkinje cells. Characteristically, mTORC1 signaling is decreased in response to cellular stressors [reviewed in 27]. That either depression or enhancement of mTORC1 signaling can underlie Purkinje cell impairment was previously shown in mice where mTORC1 signaling was decreased due to a loss of mTORC1 or in Purkinje cells in which mTORC1 signaling was enhanced due to the absence of the mTORC1 inhibitor *TSC1*, the function as well as survival of Purkinje cells were adversely affected [28]. It is intriguing to speculate that, as seen with mTORC1 signaling levels in cerebella of *Pcp2*-*ATXN1[82Q]* and *Pcp2*-*ATXN2[127Q]* transgenic mice, the SCAs might also be categorized according to mTORC1 signaling activity with SCA1 having reduced Purkinje cell mTORC1 signaling and SCA2 showing enhanced Purkinje cell mTORC1 signaling. Importantly, activation of the CCK1R in both instances restored mTORC1 signaling to a normal level. Understanding the mechanism and cellular pathways by which CCK1R activation in *Pcp2*-*ATXN1[82Q]* and *Pcp2*-*ATXN2[127Q]* transgenic Purkinje cells restores mTORC1 signaling to a normal level is an area of considerable importance regarding the potential of CCK1R activation as a therapeutic target in the SCAs.

We suggest that the results reported in our studies [12, 20], support a model where a CCK/CCK1R/mTORC1 pathway enables Purkinje cells to adapt to stress. As Golgi discovered many years ago, Purkinje cell axon recurrent collaterals form synaptic connections between Purkinje cell [29] that have a role in modulating synchronized Purkinje cell firing [30]. Perhaps these recurrent Purkinje cell synapses also provide the pathway by which the release of CCK peptide by Purkinje cells activates CCK1R/mTORC1 pathway in an autocrine fashion.

In the case of ATXN1[82Q] expressing Purkinje cells, cumulative stress induced by mutant ATXN1 promotes the cleavage of CCK to the octapeptide CCK-8, the natural ligand with the highest affinity for Cck1R [31] that upon secretion binds to and activates CCK1R on Purkinje cells. In addition, expanded ATXN1 reduces CCK expression, thus dampening the ability of Purkinje cells to respond to stress and promoting ATXN1 pathogenesis. Activation of Purkinje cell CCK1R stimulates mTORC1, which we speculate is a critical component by which CCK1R activation dampens the pathogenic effects of expanded ATXN1. Inactivation of mTORC1 induces a progressive loss of Purkinje cells by apoptosis [28]. mTORC1 signaling, in addition to responding to many stresses, is impaired in *Atxn1*^*154Q/2Q*^ knock-in mice, and the absence of mTORC1 in Purkinje cells of *Atxn1*^*154Q/2Q*^ mice worsens disease [25]. We are intrigued with the report that impaired striatal mTORC1 activity underlies degenerative phenotypes in a Huntington’s disease mouse model brain and activation of mTORC1 alleviates striatal atrophy [32]. Perhaps, manipulation of the CCK-CCK1R pathway might be a therapeutic target for treating neurodegenerative diseases involving other neurons as well as Purkinje cells.
